# Supplementary material for: COZOID: contact zone identifier for visual analysis of protein-protein interactions
Source: BMC Bioinformatics. 2018 Apr 6;19:125. doi: 10.1186/s12859-018-2113-6 (PMC5889581; doi:10.1186/s12859-018-2113-6)
Supplement: Supplementary file 2 — Software build. Executable binary file of the software tool. (ZIP 118,784 kb) [file 12859_2018_2113_MOESM2_ESM.zip › cozoid/java/docs/beansbinding-1.2.1-doc.zip!/org/jdesktop/beansbinding/AutoBinding.html]

AutoBinding (Beans Binding)


---


|  |  |  |
| --- | --- | --- |
| **Overview** \| **Package** \| **Class** \| **Use** \| **Tree** \| **Deprecated** \| **Index** \| **Help** | |  |
| **PREV CLASS**   **NEXT CLASS** | **FRAMES**    **NO FRAMES**     **All Classes** |  |
| SUMMARY: NESTED \| FIELD \| CONSTR \| METHOD | DETAIL: FIELD \| CONSTR \| METHOD |  |


---


## org.jdesktop.beansbinding Class AutoBinding<SS,SV,TS,TV>

```
java.lang.Object
  org.jdesktop.beansbinding.Binding<SS,SV,TS,TV>
      org.jdesktop.beansbinding.AutoBinding<SS,SV,TS,TV>
```

**Type Parameters:**: `SS` - the type of source object: `SV` - the type of value that the source property represents: `TS` - the type of target object: `TV` - the type of value that the target property represents

**Direct Known Subclasses:**: JComboBoxBinding, JListBinding, JTableBinding

---

``` public class AutoBinding<SS,SV,TS,TV> extends Binding<SS,SV,TS,TV> ```

An implementation of `Binding` that automatically syncs the source
and target by refreshing and saving according to one of three update
strategies. The update strategy is specified for an `AutoBinding`
on creation, and is one of:

- `AutoBinding.UpdateStrategy.READ_ONCE`
- `AutoBinding.UpdateStrategy.READ`
- `AutoBinding.UpdateStrategy.READ_WRITE`

The behavior of `AutoBinding` for each
of the update strategies is defined as follows:

|  |  |  |
| --- | --- | --- |
| **`READ_ONCE`** |  | **Summary:**  Tries to sync the target from the source only once, at bind time. **Details:**  At bind time, tries to sync the target from the source, by calling `refreshAndNotify`. No further automatic syncing is done. |
|  | | |
| **`READ`** |  | **Summary:**  Tries to keep the target in sync with the source. **Details:**  At bind time, tries to sync the target from the source, by calling `refreshAndNotify`. Then automatically tries to sync the target from the source by calling `refreshAndNotify` when either the source changes value, or the target changes from unwriteable to writeable. |
|  | | |
| **`READ_WRITE`** |  | **Summary:**  Tries to keep both the source and target in sync with each other. **Details:**  At bind time, first tries to sync the target from the source, by calling `refresh`. If the call succeeds, notifies the binding listeners of a successful sync. If the call returns failure, then tries to instead sync the source from the target by calling `save`. If this second call succeeds, notifies the binding listeners of a succesful sync. If it returns failure, notifies the binding listeners of a failed sync, indicating the reason for the original refresh failure. Automatically responds to changes in the state of the source as follows: If the change represents a value change, use the try-refresh-then-save procedure mentioned above. Otherwise, if the change represents the source becoming writeable, tries to update the source from the target by calling `saveAndNotify`. Automatically responds to changes in the state of the target as follows: If the change represents the target simply becoming writeable, try to sync the target from the source by calling `refreshAndNotify`. If the change represents the target becoming writeable and the value changing together, use the try-refresh-then-save procedure mentioned above. Finally if the change represents the target's value changing alone, first try to sync the source from the target by calling `save`. If that succeeds, notify the listeners of a successful sync. If it returns failure due to conversion or validation, notify the listeners of a sync failure, providing the conversion or validation failure. If it fails for any other reason, then instead try to sync the target from the source by calling `refresh`. If this succeeds, notify the listeners of successful sync. Otherwise notify them of failure with the reasons for the original save failure. |

---

| **Nested Class Summary** | |
| --- | --- |
| `static class` | `AutoBinding.UpdateStrategy`             An enumeration representing the possible update strategies of an `AutoBinding`. |

**Nested classes/interfaces inherited from class org.jdesktop.beansbinding.Binding**

`Binding.SyncFailure, Binding.SyncFailureType, Binding.ValueResult<V>`


| **Constructor Summary** | |
| --- | --- |
| `protected` | `AutoBinding(AutoBinding.UpdateStrategy strategy, SS sourceObject, Property<SS,SV> sourceProperty, TS targetObject, Property<TS,TV> targetProperty, java.lang.String name)`             Create an instance of `AutoBinding` between two properties of two objects, with the given update strategy. |


| **Method Summary** | |
| --- | --- |
| `protected  void` | `bindImpl()`             Called by `Binding.bind()` to allow subclasses to initiate binding. |
| `AutoBinding.UpdateStrategy` | `getUpdateStrategy()`             Returns the `AutoBinding's` update strategy. |
| `protected  java.lang.String` | `paramString()`             Returns a string representing the internal state of the `Binding`. |
| `protected  void` | `sourceChangedImpl(PropertyStateEvent pse)`             Called to indicate that the source property has fired a `PropertyStateEvent` to indicate that its state has changed for the source object. |
| `protected  void` | `targetChangedImpl(PropertyStateEvent pse)`             Called to indicate that the target property has fired a `PropertyStateEvent` to indicate that its state has changed for the target object. |
| `protected  void` | `unbindImpl()`             Called by `Binding.unbind()` to allow subclasses to uninitiate binding. |

**Methods inherited from class org.jdesktop.beansbinding.Binding**

`addBindingListener, addPropertyChangeListener, addPropertyChangeListener, bind, bindUnmanaged, firePropertyChange, getBindingListeners, getConverter, getName, getPropertyChangeListeners, getPropertyChangeListeners, getSourceNullValue, getSourceObject, getSourceProperty, getSourceUnreadableValue, getSourceValueForTarget, getTargetNullValue, getTargetObject, getTargetProperty, getTargetValueForSource, getValidator, isBound, isManaged, isSourceUnreadableValueSet, notifySynced, notifySyncFailed, refresh, refreshAndNotify, refreshAndNotifyUnmanaged, refreshUnmanaged, removeBindingListener, removePropertyChangeListener, removePropertyChangeListener, save, saveAndNotify, saveAndNotifyUnmanaged, saveUnmanaged, setConverter, setManaged, setSourceNullValue, setSourceObject, setSourceObjectUnmanaged, setSourceProperty, setSourceUnreadableValue, setTargetNullValue, setTargetObject, setTargetObjectUnmanaged, setTargetProperty, setValidator, throwIfBound, throwIfManaged, throwIfUnbound, toString, unbind, unbindUnmanaged, unsetSourceUnreadableValue`

**Methods inherited from class java.lang.Object**

`clone, equals, finalize, getClass, hashCode, notify, notifyAll, wait, wait, wait`

**Constructor Detail**

### AutoBinding

```
protected AutoBinding(AutoBinding.UpdateStrategy strategy,
                      SS sourceObject,
                      Property<SS,SV> sourceProperty,
                      TS targetObject,
                      Property<TS,TV> targetProperty,
                      java.lang.String name)
```

:   Create an instance of `AutoBinding` between two properties of two objects,
    with the given update strategy.

    **Parameters:**: `strategy` - the update strategy: `sourceObject` - the source object: `sourceProperty` - a property on the source object: `targetObject` - the target object: `targetProperty` - a property on the target object: `name` - a name for the `Binding` **Throws:**: `java.lang.IllegalArgumentException` - if the source property or target property is `null`


**Method Detail**

### getUpdateStrategy

```
public final AutoBinding.UpdateStrategy getUpdateStrategy()
```

:   Returns the `AutoBinding's` update strategy.

    :   **Returns:**: the update strategy

---


### bindImpl

```
protected void bindImpl()
```

:   **Description copied from class: `Binding`**
:   Called by `Binding.bind()` to allow subclasses to initiate binding.
    Subclasses typically need not install `PropertyStateListeners`
    on the source property and target property as they will be notified
    by calls to `Binding.sourceChangedImpl(org.jdesktop.beansbinding.PropertyStateEvent)` and `Binding.targetChangedImpl(org.jdesktop.beansbinding.PropertyStateEvent)`
    when the source and target properties change respectively.

    :   **Specified by:**: `bindImpl` in class `Binding<SS,SV,TS,TV>`
    :   **See Also:**: `Binding.unbindImpl()`

---


### unbindImpl

```
protected void unbindImpl()
```

:   **Description copied from class: `Binding`**
:   Called by `Binding.unbind()` to allow subclasses to uninitiate binding.

    :   **Specified by:**: `unbindImpl` in class `Binding<SS,SV,TS,TV>`
    :   **See Also:**: `Binding.bindImpl()`

---


### paramString

```
protected java.lang.String paramString()
```

:   Returns a string representing the internal state of the `Binding`.
    This method is intended to be used for debugging purposes only,
    and the content and format of the returned string may vary between
    implementations. The returned string may be empty but may not
    be `null`.

    :   **Overrides:**: `paramString` in class `Binding<SS,SV,TS,TV>`
    :   **Returns:**: a string representing the state of the `Binding`.

---


### sourceChangedImpl

```
protected void sourceChangedImpl(PropertyStateEvent pse)
```

:   **Description copied from class: `Binding`**
:   Called to indicate that the source property has fired a
    `PropertyStateEvent` to indicate that its state has changed for
    the source object. Called after the `Binding` has notified
    any property change listeners and `BindingListeners` that
    the source value has been edited (only if the `PropertyStateEvent`
    represents a value change). This method is useful for subclasses
    to detect source changes and perform syncing as appropriate.

    :   **Overrides:**: `sourceChangedImpl` in class `Binding<SS,SV,TS,TV>`

---


### targetChangedImpl

```
protected void targetChangedImpl(PropertyStateEvent pse)
```

:   **Description copied from class: `Binding`**
:   Called to indicate that the target property has fired a
    `PropertyStateEvent` to indicate that its state has changed for
    the target object. Called after the `Binding` has notified
    any property change listeners and `BindingListeners` that
    the target value has been edited (only if the `PropertyStateEvent`
    represents a value change). This method is useful for subclasses
    to detect target changes and perform syncing as appropriate.

    :   **Overrides:**: `targetChangedImpl` in class `Binding<SS,SV,TS,TV>`


---


|  |  |  |
| --- | --- | --- |
| **Overview** \| **Package** \| **Class** \| **Use** \| **Tree** \| **Deprecated** \| **Index** \| **Help** | |  |
| **PREV CLASS**   **NEXT CLASS** | **FRAMES**    **NO FRAMES**     **All Classes** |  |
| SUMMARY: NESTED \| FIELD \| CONSTR \| METHOD | DETAIL: FIELD \| CONSTR \| METHOD |  |


---
